# Critical thinking in early medical education: experiences from a first-year science course

**DOI:** 10.3389/fmed.2026.1816516

**Published:** 2026-05-04

**Authors:** Moteeb Alhamidani

**Affiliations:** 1 King Abdullah International Medical Research Center, Riyadh, Saudi Arabia; 2 King Saud bin Abdulaziz University for Health Sciences, Riyadh, Saudi Arabia; 3 Ministry of National Guard - Health Affairs, Riyadh, Saudi Arabia

**Keywords:** critical thinking, first-year medical students, higher education, medical education, qualitative research, threshold concepts

## Abstract

**Introduction:**

Critical thinking is emphasized as an important learning aim in early medical education, yet how students experience learning this capability in practice remains underexplored. Understanding students’ experiences of learning critical thinking provides insight into how educational intentions are encountered rather than requiring measurement of whether learning has occurred.

**Methods:**

This study explored how first-year medical students experienced learning critical thinking in a science course. An interpretive qualitative design with reflexive thematic analysis and semi-structured interviews was used. Twelve first-year medical students participated.

**Results:**

Analysis generated four themes: navigating epistemic uncertainty about what critical thinking meant in context; engaging in active analytical practice through specific course activities; inhabiting a threshold of difficulty marked by persistent discomfort and evaluative ambiguity; and experiencing affective and identity-level responses to a cognitively demanding new standard of learning. Students reported that learning critical thinking felt different from other forms of learning they had previously encountered, often more demanding and less straightforward. Course activities such as problem-solving exercises and evaluating evidence were experienced as opportunities to practice critical thinking, though students varied in how comfortable or successful they felt during these activities. Moments of difficulty were common and included struggling to know how much analysis was sufficient or feeling uncertain about whether their thinking was appropriately critical.

**Discussion:**

The findings indicate that students experience learning critical thinking as a complex process involving cognitive effort, emotional engagement, and ongoing interpretation of what is being asked. Attending to how students experience this learning provides a perspective on early medical education that complements outcome-focused approaches.

## Introduction

Critical thinking is widely identified as an essential competency in medical education, supporting clinical reasoning, decision-making, and professional judgment (1, 2). However, critical thinking is also widely recognized as a contested construct in higher education, with definitions varying across skill-focused and disposition-focused accounts (3, 4). For clarity in the present study, “critical thinking” is used in the broad educational sense of purposeful analysis and evaluation of information and arguments to reach justified judgments (3, 5). Institutions emphasize critical thinking in course descriptions and learning outcomes, particularly in early medical training where foundational cognitive skills are developed (6). Reviews of critical thinking pedagogy also note that institutional emphasis often coexists with limited explicit instruction in critical thinking processes, as teaching approaches may prioritize subject content over critical thinking development (3).

Although critical thinking is frequently named as a learning aim, how students experience engaging with this aim in practice is less well understood (7). One reason for this gap is that students’ experiences of learning encompass not only what they do but also how they interpret learning activities, how they feel during learning, and how they make sense of educational expectations (8, 9). Understanding these experiential dimensions provides insight into how educational intentions are encountered from the learner’s perspective, which is distinct from measuring whether learning outcomes have been achieved (10).

First-year science courses in medical education often serve as contexts where critical thinking is explicitly emphasized. Such courses typically combine content mastery with development of analytical capabilities, requiring students to both acquire knowledge and learn to think differently about information (11, 12). Students entering these courses bring prior learning experiences that may not have emphasized critical thinking in the same ways, creating a transition that can feel challenging or unfamiliar (13, 14). Research in nursing education has similarly explored how students experience learning various competencies, finding that students’ subjective experiences of difficulty, engagement, and sense-making are important aspects of the learning process (15, 16). Likewise, examining how students experience learning critical thinking, including what activities feel meaningful and what aspects feel demanding, can inform understanding of early medical education (17).

Exploring how students experience learning critical thinking is valuable because it attends to the lived reality of engaging with this educational aim. Two theoretical perspectives help frame this inquiry. First, epistemic cognition theory focuses on how learners understand the nature, limits, and justification of knowledge—a dimension directly implicated when students must evaluate arguments and resist simple certainty (18, 19). Students with less sophisticated epistemic beliefs may struggle with the open-endedness of critical thinking tasks, experiencing ambiguity as threatening rather than intellectually productive. Second, threshold concepts theory proposes that certain disciplinary ideas function as conceptual gateways: once genuinely understood, they are transformative and irreversible, but before understanding occurs they can produce a “stuck place” of confusion and discomfort (20). Critical thinking shares several defining properties of threshold concepts—it is troublesome relative to prior learning habits, potentially transformative in how students relate to knowledge, and integrative across domains—and may therefore function as a threshold concept in early medical education. Together these frameworks suggest that students’ experiences of learning critical thinking are not simply motivational or affective events but are epistemically and conceptually structured encounters. When students describe their experiences, they reveal how learning activities are interpreted, what emotional responses accompany cognitive work, and where they find meaning or encounter confusion (21, 22). Unlike outcome-based approaches, experience-focused inquiry does not require demonstration of learning effectiveness. Instead, it examines how students encounter and navigate educational contexts, providing a different form of insight into educational processes than traditional outcome measurement (7, 10). Studies examining student experiences in health professions education have noted that attending to experiential accounts can highlight aspects of learning that are not visible through performance data alone, such as the sense-making work and the affective dimensions involved in demanding cognitive tasks (23, 24). Ultimately, understanding how students experience learning critical thinking offers a perspective on first-year medical education that emphasizes the learner’s subjective encounter with course aims (25).

This study aimed to explore how first-year medical students experienced learning critical thinking in a science course.

## Methods

This study employed a qualitative design consistent with interpretive description, a methodology developed specifically for applied health education professions research that aims to generate clinically and educationally useful knowledge through interpretive engagement with participants’ experiences (26). Interpretive description is grounded in an interpretivist epistemological position, which holds that meaning is constructed through individuals’ subjective experiences and that understanding those experiences requires interpretive engagement rather than detached observation (27). This epistemological stance is consistent with reflexive thematic analysis (RTA), which was used as the primary analytic method to identify patterns in how students described their experiences. As Braun and Clarke (28, 29) position it, RTA is a theoretically flexible but not a theoretical method; it is interpretive rather than purely descriptive, generating themes through active researcher engagement with the data rather than extracting pre-existing truths. Accordingly, this study aimed to develop an interpretive account of students’ experiential encounters with learning critical thinking, rather than to produce a low-inference descriptive summary of events. The method follows iterative phases of data familiarization, initial coding, theme development, and refinement, and was selected because it allows detailed exploration of experiential accounts while remaining grounded in participants’ own language and perspectives.

Twelve first-year medical students participated in the study. All were enrolled in a science course that explicitly identified critical thinking as a learning aim. The course ran throughout the fall semester and included lectures, problem-solving sessions, small-group discussions, and case-based activities. These active-learning components are consistent with pedagogies commonly used to support critical thinking practice, such as problem-based learning and project-based learning that emphasize authentic problems, collaborative discussion, and feedback (5, 30). Critical thinking was assessed in the course through a combination of methods: written case analysis assignments in which students were required to evaluate competing scientific explanations and justify their reasoning with evidence; an end-of-course reflective essay in which students articulated their approach to analytical problem-solving; and examination questions that included open-ended data interpretation and argument evaluation tasks scored using a rubric that rewarded evidence-based reasoning alongside factual accuracy. This assessment context is relevant because it shaped the particular pressures students described, including uncertainty about whether their analytical efforts would be recognized in grading. Purposive sampling was used to recruit students willing to reflect on their learning experiences. Students were invited to participate near the end of the semester, allowing them to have experienced the full course while memories of their engagement remained accessible. The rationale for the sample size of 12 is addressed in the analysis section below. Demographic information was kept minimal to preserve confidentiality.

Semi-structured interviews lasting 35–50 min were conducted with each participant. The interview guide included questions about how students would describe their experience of learning critical thinking in the course, what activities or moments stood out as meaningful or significant, how learning critical thinking felt compared to other learning they had done, and what emotional or cognitive responses they noticed during the learning process. Students were encouraged to describe specific instances and to reflect on both positive and challenging aspects of their experience. All interviews were audio-recorded and transcribed verbatim.

Transcripts were analyzed using reflexive thematic analysis following the phases described by Braun and Clarke (28). In keeping with guidance on reporting reflexive thematic analysis, themes were treated as interpretive outputs generated through systematic engagement with the dataset rather than as entities that passively “emerge” (29). Analysis began with repeated reading to develop familiarity with students’ experiential accounts. Initial codes captured different aspects of how students described their experiences of learning critical thinking. Codes were organized into candidate themes representing distinct experiential dimensions. Themes were reviewed and refined through iterative critical reflection—returning repeatedly to the coded data to test whether candidate themes were internally coherent, sufficiently distinct, and grounded in participants’ language—to ensure they captured the richness of students’ descriptions without reducing experience to simple categories.

Throughout refinement, the author engaged reflexively with how his own assumptions about “critical thinking” might shape coding decisions, to strengthen analytic coherence and transparency (29). The sample size of 12 was considered adequate based on principles of information power: a sample provides sufficient information power when the aim is narrow, the theory is specific, the participants are homogeneous, the analysis strategy attends to the full dataset rather than individual cases, and the dialogue quality is dense (31). All five conditions were met in the present study—the aim was bounded (experiences of one course), the theoretical framework was specified in advance, participants were drawn from a single cohort, and interviews were in-depth and richly detailed—supporting the adequacy of the sample without claiming saturation.

Reflexivity was treated as an ongoing methodological responsibility throughout the study. As the sole author, the researcher held an educational role within the institution where the study was conducted and had prior experience teaching in medical education contexts. This positionality meant that assumptions about what “good” critical thinking pedagogy looks like, and what difficulties students “should” encounter, functioned both as an analytic resource and a potential source of interpretive bias. To manage this, the researcher maintained a reflexivity journal throughout data collection and analysis, recording decisions, interpretive choices, and instances where initial readings aligned too neatly with prior expectations.

Ethical approval was obtained from the institutional review board, and all participants provided informed consent. Confidentiality was maintained through anonymization of all identifying information.

## Results

Students described experiencing learning critical thinking through four interrelated themes. These themes reflected: (1) navigating epistemic uncertainty—the ongoing work of interpreting what critical thinking meant and what counted as adequate analysis; (2) engaging in active analytical practice—encountering specific activities as sites of critical thinking development; (3) inhabiting a threshold of difficulty—experiencing a liminal space of persistent discomfort and uncertainty characteristic of working toward conceptual transformation; and (4) affective and identity-level responses—the emotional and self-evaluative dimensions of encountering a cognitively demanding new standard of learning. Together, these themes illustrate the complexity of students’ experiential encounter with critical thinking as a learning aim. The findings from each theme are described below.

Students described working to understand what critical thinking really meant in the context of the course—what this study frames as navigating epistemic uncertainty. Initially, although the term was used frequently, its meaning was not immediately clear to them. Participant 2 reflected, “I heard ‘critical thinking’ over and over but it took me a while to understand what they actually wanted us to do.” Participant 7 described a similar process of gradual inference: “I kept watching which answers the instructor praised and which ones she said needed more depth—that’s how I started to figure out what critical thinking looked like.” Participant 11 noted that the ambiguity persisted well into the course: “Even by the middle of the semester I was not fully sure if I was doing it right. I kept second-guessing my own analysis.” They attempted to make sense of what counted as critical thinking by observing which activities instructors labelled as such and by paying attention to feedback on their work. Over time, some students came to understand critical thinking as involving questioning, evaluating evidence, or considering multiple perspectives, while others associated it more with analytical reasoning or systematic problem-solving. This sense-making process was ongoing rather than resolved at a single point, with students continuing to refine their understanding as the course progressed. In fact, the very act of interpreting what critical thinking meant was itself part of how students experienced learning it—a process consistent with the epistemic work required when learners encounter a genuinely new standard of knowledge use.

Students described engaging with specific activities that they experienced as opportunities for active analytical practice. Problem-solving exercises, case analyses, and activities requiring evaluation of evidence were frequently mentioned as contexts where critical thinking felt actively engaged. Participant 4 noted, “When we had to figure out which explanation made the most sense based on the data, that felt like critical thinking.” Participant 9 described the case analysis format as particularly generative: “The cases pushed you to not just remember facts but to ask why—why this diagnosis and not that one, why this evidence is stronger.” Participant 1 captured the contrast with passive learning: “In lectures I could just write things down, but in the problem sessions I actually had to decide something and defend it.” Students varied in how they experienced these activities, with some finding them engaging and intellectually stimulating while others found them frustrating or anxiety-provoking. Group discussions were experienced as valuable by some students who appreciated hearing others’ thinking processes, while other students found group work less helpful if participants engaged unevenly. The experience of engaging with these activities was shaped both by the activity design and by students’ comfort with the type of thinking required. Students noted that these activities felt different from lectures or reading assignments, requiring more active cognitive participation.

Students described encountering moments of uncertainty and difficulty as part of learning critical thinking—a pattern this study frames as inhabiting a threshold of difficulty. Several noted that critical thinking tasks often lacked clear right answers, which created discomfort. Participant 6 explained, “With memorization you know if you got it right, but with critical thinking it’s less clear whether your analysis is good enough.” Participant 10 expressed a similar sense of evaluative uncertainty: “I would finish a task and still not know if I had actually been critical or just thorough. There is a difference and I could not always tell.” Participant 3 described the difficulty of questioning authoritative sources: “It felt strange to question what the textbook said. I kept thinking, who am I to disagree with this? But that was actually what the course wanted.” Students described struggling to know when they had analyzed something sufficiently or how to tell if their reasoning was sound. Uncertainty about assessment criteria added to this difficulty, as students worried whether their efforts would be recognized in grading. Some students experienced difficulty when trying to question information that seemed authoritative, finding it hard to adopt an evaluative stance toward textbook content or instructor explanations. These moments of difficulty were described as frustrating but also sometimes as productive, prompting deeper engagement with material. Students’ experiences of difficulty were not uniform, with some finding uncertainty stimulating and others finding it stressful.

Students described varied affective and identity-level responses to learning critical thinking. Several noted that critical thinking required more mental effort than other forms of learning, feeling more cognitively demanding. Participant 5 reflected, “It’s tiring in a different way, because you have to really think hard instead of just absorbing information.” Participant 8 described a shift in how they saw themselves as a learner: “I started to feel like maybe I was not as smart as I thought, because things that used to be easy—like studying and getting a grade—did not work the same way here.” Participant 12 expressed how emotional responses were entangled with evaluative uncertainty: “When I could not tell if my thinking was good, I felt anxious. It was not just hard—it made me question whether I belonged in medicine.” This last account reflects a dimension of belonging uncertainty that has been documented in early medical training, where cognitive challenges can interact with identity formation. Students described experiencing satisfaction when they successfully worked through analytical tasks, but also frustration when progress felt slow or when they struggled to apply critical thinking skills. Some students experienced anxiety about whether they were developing critical thinking adequately or whether they would be able to demonstrate it on assessments. Others described curiosity and intellectual engagement when encountering problems that required careful reasoning. The affective dimension of learning critical thinking was prominent in students’ accounts, with emotional responses intertwined with cognitive experiences and, for some, with questions about their own academic identity. Students noted that their responses varied across different activities and different points in the semester, suggesting that experience was not static but shifted over time.

## Discussion

Students in this study experienced learning critical thinking as a complex process organized around four interrelated themes: navigating epistemic uncertainty about what critical thinking required; engaging in active analytical practice through course activities; inhabiting a threshold of difficulty marked by persistent discomfort and evaluative ambiguity; and experiencing affective and identity-level responses to a new cognitive standard. Together these themes, interpreted through the lenses of epistemic cognition theory (19) and threshold concepts theory (20), illustrate how students encountered critical thinking as a learning aim during their first year of medical education (2, 7). The first theme—navigating epistemic uncertainty—reflects the challenge of developing more sophisticated beliefs about knowledge in a context where the standard for “good” thinking was not self-evident (6, 9). This interpretive work is unsurprising given that critical thinking is widely described as difficult to define consistently in higher education, and students may be navigating multiple, sometimes implicit, conceptions of what counts as “critical” work (3, 4). Research with international higher education students further suggests that psychological and sociocultural factors—including confirmation bias, framing effects, and prior educational traditions that reward certainty—can impede critical thinking and reduce learners’ tolerance of ambiguity (4). In other words, students did not simply receive a definition of critical thinking to apply; rather, they worked to understand what was being asked of them by engaging with course activities and attending to feedback (21, 22). This reliance on feedback is consistent with intervention research showing that scaffolding and feedback within active learning course designs can strengthen critical thinking development (5).

This interpretive work is itself an important aspect of the learning experience, highlighting that engaging with educational aims requires cognitive effort beyond mere task completion (8). Viewed through the lens of epistemic cognition theory, students’ struggle to define and apply critical thinking reflects the challenge of developing more sophisticated beliefs about knowledge: moving from seeing learning as the accumulation of right answers toward understanding that knowledge can be contested, constructed, and evaluated (19). Similarly, from a threshold concepts perspective, students’ accounts of a prolonged “stuck place”—where expectations were unclear and difficulty felt unresolvable—are consistent with the liminal space that learners inhabit before a threshold concept is genuinely traversed (20). It is worth noting that critical thinking shares key properties of threshold concepts as described by Meyer and Land: it is troublesome (counterintuitive relative to rote learning), transformative (it changes how students relate to knowledge), and potentially irreversible once genuinely understood. This framing helps explain why sense-making was not resolved at a single point but remained ongoing throughout the course. The affective and identity-level responses students described—including self-doubt, anxiety about performance, and questions about belonging—are also theoretically coherent within this framework: the emotional turbulence of the liminal state is a recognized feature of threshold concept traversal, not merely a motivational or anxiety response (20). The variation in how students described experiencing particular activities, with some finding them engaging and others finding them difficult, further underscores individual differences in epistemic development and prior preparation (14, 16). Recognizing these varied experiences emphasizes the subjective nature of how students encounter educational contexts, rather than assuming uniform experience across all learners (4).

Students’ experiences were also shaped by the learning context, including specific course activities, instructional approaches, and assessment structures. For example, activities that required active analytical work, such as problem-solving exercises or evidence evaluation, were experienced as opportunities to engage with critical thinking, though the quality of that experience varied (11, 12). When activities were well-designed and clearly tied to course objectives, students had more positive learning experiences, whereas unclear or misaligned activities often led to confusion (17, 32). The uncertainty students experienced about assessment criteria shaped their emotional responses to learning, with anxiety arising when they could not discern how their critical thinking would be evaluated (15, 24). This finding is consistent with research in health professions education indicating that assessment clarity influences student experience (24). The difficulty students encountered, especially around open-ended tasks lacking clear right answers, reflects inherent features of critical thinking rather than mere flaws in the course design (10, 13). However, the way such difficulty is framed and supported does influence whether students perceive it as productive challenge or as overwhelming obstacle. In sum, these findings suggest that careful attention to course design and instructional clarity may improve how students experience learning critical thinking.

Examining how students experience learning critical thinking provides a value distinct from simply measuring learning outcomes. Students’ firsthand accounts reveal the lived complexity of engaging with demanding cognitive tasks, including the interpretive work, emotional responses, and encounters with difficulty that characterize critical thinking (21, 22). Many of these aspects of experience are not captured by performance-based measures, yet they remain important dimensions of educational engagement (10, 25). Focusing on experience rather than solely on effectiveness allows for understanding how students navigate educational transitions without requiring demonstration of achievement (4). This approach recognizes students’ perspectives as legitimate sources of insight into educational processes, rather than viewing experience as merely subjective impression requiring verification (23). The findings from this study suggest that students’ experiences of learning critical thinking are rich and varied, shaped by both individual factors and contextual features, and that these experiences are worthy of attention in their own right. Understanding the student experience thus complements outcome-focused research by providing a different window into early medical education, one that attends to how learning feels and what it means to students facing new cognitive demands.

The findings point to several practical steps for supporting students’ experiences of learning critical thinking. First, making explicit what ‘critical thinking’ means in specific course contexts may reduce the interpretive burden on students and allow them to focus cognitive effort on developing skills instead of deciphering expectations. For example, structured problem-based learning tasks can make reasoning processes visible and help students persist through productive struggle (30). Second, designing learning activities that clearly target critical thinking practice, while also offering appropriate support and feedback, may enhance students’ engagement. In online or blended formats, scaffolds that support collaborative regulation and feedback cycles may further strengthen critical thinking practice (5). Third, openly acknowledging that learning critical thinking involves difficulty and uncertainty, and framing these challenges as normal rather than as signs of inadequacy, may help students approach obstacles more productively. Explicit teaching about common reasoning pitfalls (e.g., confirmation bias and framing effects) may also increase students’ metacognitive awareness and tolerance of ambiguity during critical thinking tasks (4). Fourth, providing clarity about how critical thinking will be assessed may reduce anxiety and allow students to direct their efforts more confidently. Finally, creating space for students to discuss their learning experiences, including what they find challenging or unclear, may support their sense-making and help them realize that varied responses to learning are common. Overall, these implications focus on bolstering students’ experience of learning critical thinking rather than prescribing any single pedagogical approach.

## Limitations

This study has several limitations. First, its small sample size (*n* = 12) and single-institution context limit transferability of findings. Second, the study relied on students’ retrospective reflections on their experiences, which may have been influenced by memory lapses or by events that occurred later in the course. Third, students’ accounts represent subjective interpretations of their learning experience rather than objective records of what actually happened. Fourth, the study did not assess learning outcomes, nor did it examine how the described experiences corresponded to actual performance or skill development. Finally, the findings describe how a specific group of students experienced learning in a specific context and should not be generalized to all first-year medical students or to all courses emphasizing critical thinking.

## Future research directions

Future research should examine students’ experiences across multiple institutions and course types to explore how variations in pedagogy and assessment shape sense-making and emotional responses. Triangulating interview data with course artifacts (e.g., learning outcomes, assessment rubrics) and observations could also provide a richer account of how critical thinking is enacted in practice. Intervention studies of scaffolded problem- or project-based designs could test whether supports such as collaborative regulation scaffolding and feedback alter the kinds of uncertainty students described (5, 30). Given the increasing presence of generative AI tools in higher education, future work could explore how AI literacy and AI-supported study practices influence students’ evaluation of evidence and arguments (33, 34).

## Conclusion

This study explored how first-year medical students experienced learning critical thinking in a science course. Analysis generated four themes: navigating epistemic uncertainty about what critical thinking required; engaging in active analytical practice through specific course activities; inhabiting a threshold of difficulty characterized by evaluative ambiguity and persistent discomfort; and experiencing affective and identity-level responses to a new and demanding cognitive standard. Together, these themes illustrate the complexity of how students encounter critical thinking as a learning aim, and suggest that theoretical lenses such as epistemic cognition and threshold concepts offer productive frames for interpreting students’ experiences.

Students’ experiences of learning critical thinking were shaped by both individual interpretations and contextual features of the course. Attending to how students experience learning provides insight into the lived reality of engaging with demanding cognitive aims in early medical education. The value of understanding student experience lies not in verifying whether learning occurred, but in recognizing that students’ subjective encounters with educational intentions are meaningful in themselves.

This study contributes to understanding of early medical education by examining students’ experiential encounter with learning critical thinking. The focus on experience as distinct from outcome offers a perspective that complements effectiveness research. This experience-centered perspective may also inform more supportive approaches to help students navigate cognitive transitions in medical training.

## Data Availability

The raw data supporting the conclusions of this article will be made available by the authors, without undue reservation.
